# Therapeutic efficacy of recombinant human brain natriuretic peptide in elderly chronic heart failure patients: associations with changes in PⅢNP and suPAR levels

**DOI:** 10.3389/fcvm.2026.1866308

**Published:** 2026-09-25

**Authors:** Liangdong Lv, Peng Zhou, Fanqi Kong, Ruifang Song

**Affiliations:** 1 Department of Cardiology, The Third Affiliated Hospital of Wenzhou Medical University, Ruian, China; 2 Department of Electrocardiogram Function, The Third Affiliated Hospital of Wenzhou Medical University, Ruian, China; 3 Department of Nephrology, The Third Affiliated Hospital of Wenzhou Medical University, Ruian, China

**Keywords:** biomarkers, elderly, natriuretic activity, peptide, suPAR

## Abstract

**Objective:**

To systematically evaluate the clinical efficacy of recombinant human brain natriuretic peptide (rhBNP) added to conventional treatment in elderly patients with chronic heart failure (CHF), and to analyze its regulatory effects on fibrosis- and inflammation-related biomarkers such as procollagen type III amino-terminal propeptide (PⅢNP) and soluble urokinase-type plasminogen activator receptor (suPAR).

**Methods:**

A retrospective comparative cohort study was conducted to collect clinical data of 124 elderly CHF patients treated in our hospital from February 2023 to April 2025. According to the treatment regimens received, the patients were divided into two groups: the control group (*n* = 62) received conventional treatment, while the study group (*n* = 62) received rhBNP in addition to conventional treatment. The clinical therapeutic effects, cardiac function indicators [left ventricular ejection fraction (LVEF), left ventricular end-diastolic diameter (LVEDD), left ventricular end-systolic diameter (LVESD), cardiac index (CI)], blood gas indices [arterial oxygen partial pressure (PaO2), arterial carbon dioxide partial pressure (PaCO2)], myocardial injury markers [N-terminal pro-B-type natriuretic peptide (NT-proBNP), cardiac troponin I (cTnI), lactate dehydrogenase (LDH), creatine kinase-MB (CK-MB)], fibrosis and inflammation-related biomarkers (PⅢNP, suPAR), as well as adverse reactions and adverse prognosis, were compared between the two groups.

**Results:**

(1) Clinical therapeutic effects: Post-treatment, the overall efficacy of the study group was significantly better than that of the control group (total effective rate: 93.55% vs. 82.26%, *P* < 0.05). (2) Cardiac function indicators: Post-treatment, LVEF and CI levels increased compared with Pre-treatment, while LVEDD and LVESD decreased in both groups, with greater changes observed in the study group (*P* < 0.05). (3) Blood gas indices: Post-treatment, PaO2 increased and PaCO2 decreased in both groups compared with Pre-treatment, with greater changes observed in the study group (*P* < 0.05). (4) Myocardial injury markers: Post-treatment, NT-proBNP, cTnI, LDH, and CK-MB levels decreased in both groups compared with Pre-treatment, with greater reductions in the study group (*P* < 0.05). (5) Fibrosis and inflammation-related biomarkers: Post-treatment, PⅢNP and suPAR levels decreased in both groups compared with Pre-treatment, with more pronounced improvements observed in the study group (*P* < 0.05). (6) Adverse reactions and adverse prognosis: There were no statistically significant differences between the two groups in the incidence of related adverse reactions or adverse prognosis (*P* > 0.05).

**Conclusion:**

Elderly CHF patients receiving rhBNP in addition to conventional treatment showed significantly greater improvements in cardiac function, myocardial injury markers, blood gas parameters, and fibrosis- and inflammation-related biomarkers (PⅢNP, suPAR). Meanwhile, the treatment was well tolerated, with no obvious increase in adverse reactions or adverse prognosis. However, considering the limitations of the study design, these findings mainly reflect correlation rather than causation. Further validation is required through multicenter, large-sample, prospective randomized controlled trials.

## 1 Introduction

Chronic heart failure (CHF) is a complex clinical syndrome resulting from any structural or functional impairment of ventricular filling or ejection of blood, characterized by typical symptoms (such as breathlessness and fatigue) and signs (such as fluid retention and elevated natriuretic peptides) (1). CHF remains a leading cause of hospitalization and mortality, particularly among the elderly population who often present with complex comorbidities and poor responses to conventional therapies (2, 3). Based on left ventricular ejection fraction (LVEF), CHF is further categorized into heart failure with reduced ejection fraction (HFrEF, LVEF ≤ 40%), heart failure with mildly reduced ejection fraction (HFmrEF, LVEF 41%–49%), and heart failure with preserved ejection fraction (HFpEF, LVEF ≥ 50%), in accordance with the Chinese Guidelines for the Diagnosis and Treatment of Heart Failure 2024 (4). Despite guideline-directed medical therapy, clinical outcomes in this subgroup remain suboptimal, highlighting an urgent need for adjunctive treatment strategies that can further improve hemodynamics and reverse pathological remodeling (5).

Two critical drivers of CHF progression are myocardial fibrosis and systemic inflammation. Myocardial fibrosis leads to ventricular stiffness and diastolic dysfunction, and also contributes to systolic dysfunction, while chronic inflammatory activation exacerbates cardiac injury and adverse remodeling (6, 7). Procollagen type III amino-terminal propeptide (PⅢNP) and soluble urokinase-type plasminogen activator receptor (suPAR) have emerged as robust biomarkers reflecting these distinct yet interconnected pathways. PⅢNP is a propeptide released during the synthesis of type III collagen and serves as a marker of active fibrogenesis and extracellular matrix turnover; suPAR, a regulator of innate immune activity, indicates sustained immune activation and systemic inflammation (8, 9). Elevated levels of both markers are independently associated with worse prognosis in CHF patients, suggesting that targeting both fibrotic and inflammatory burdens may offer therapeutic benefits beyond standard care (10, 11).

Recombinant human brain natriuretic peptide (rhBNP) is a potent vasodilator with natriuretic and diuretic properties that reduces cardiac preload and afterload (12, 13). While rhBNP is established in the management of acute decompensated heart failure, its efficacy and mechanistic impact on long-term structural and inflammatory markers in elderly CHF patients remain unclear. Specifically, whether rhBNP exerts pleiotropic effects by attenuating myocardial fibrosis and suppressing inflammatory activation—thereby lowering PⅢNP and suPAR levels—is not well understood.

Therefore, based on the theoretical framework of BNP's pleiotropic effects, we postulated that adjunctive rhBNP therapy in elderly CHF patients is associated with improved clinical outcomes, mediated by significant reductions in fibrosis (PⅢNP) and inflammation (suPAR) burden. The primary objective of this study was to evaluate whether this association holds true in clinical practice, comparing outcomes between patients receiving rhBNP plus conventional therapy versus those receiving conventional therapy alone. Secondary objectives included assessing improvements in cardiac function parameters, myocardial injury markers, blood gas indices, and specifically investigating the modulatory effects of rhBNP on PⅢNP and suPAR levels.

## 2 Data and methods

The study was conducted in strict accordance with the principles of the Declaration of Helsinki and was approved by the Ethics Committee of the Third Affiliated Hospital of Wenzhou Medical University. As this was a retrospective study using previously collected clinical data, the Ethics Committee granted a waiver of written informed consent.

### 2.1 Study design and participants

This study was a single-center, retrospective comparative cohort study of hospitalized elderly CHF patients. Clinical data of elderly CHF patients who were hospitalized and received related treatment in our hospital from February 2023 to April 2025 were retrospectively collected and analyzed. According to the different treatment regimens received, patients who met the inclusion criteria and had complete clinical data were divided into the control group (*n* = 62, receiving conventional treatment) and the study group (*n* = 62, receiving rhBNP in addition to conventional treatment). All 124 enrolled patients successfully completed the 3-month follow-up period after discharge, with no losses to follow-up. The sample size was determined based on a two-sample independent t-test for the primary outcome (change in LVEF), with a power of 80% and a two-sided significance level of 0.05. According to previously published studies on rhBNP in elderly CHF patients (13, 14), the anticipated difference in LVEF improvement between groups was approximately 5% (standard deviation 7%). Using the formula $n=2\times (Z\alpha /2+Z\beta {)}^{2}\times \sigma^{2}/\delta^{2}$, where Z α/2 = 1.96, Z β  = 0.842 (for 80% power), σ  = 7%, and δ  = 5%, the calculated sample size was approximately 62 patients per group. Thus, the final analysis included 62 patients per group.

### 2.2 Inclusion and exclusion criteria

#### 2.2.1 Inclusion criteria

(1) Age ≥ 60 years, regardless of sex; (2) Hospitalized patients diagnosed with CHF and meeting the diagnostic criteria in the Chinese Guidelines for the Diagnosis and Treatment of Heart Failure 2024 (4), with LVEF < 50% documented by echocardiography within 48 h of admission; (3) Received conventional heart failure treatment during hospitalization or rhBNP in addition to conventional treatment, with complete treatment and follow-up data; (4) New York Heart Association (NYHA) cardiac function class III–IV (15); (5) Possessed normal communication and cognitive abilities and could cooperate with study-related procedures. Hospitalization was defined as admission for acute decompensation or worsening of CHF symptoms (worsening dyspnea, increasing peripheral edema, or reduced exercise tolerance) requiring intensive medical evaluation and intravenous diuretic or inotropic therapy, but not requiring emergency percutaneous coronary intervention, mechanical circulatory support, or emergency intubation on the day of admission.

#### 2.2.2 Exclusion criteria

(1) Patients with acute coronary syndrome or acute decompensated heart failure requiring emergency interventional treatment on the first day of admission; (2) Severe hepatic dysfunction (defined as serum alanine aminotransferase or aspartate aminotransferase > 3 times the upper limit of normal, or total bilirubin > 2 times the upper limit of normal) or severe renal dysfunction [defined as estimated glomerular filtration rate < 30 mL/min/1.73 m^2^ or serum creatinine > 3 mg/dL (265 µmol/L) or requiring renal replacement therapy]; (3) Known active inflammation or infection, active malignancy, or systemic autoimmune diseases; (4) Allergy or contraindication to drugs or tests used in the study; (5) Missing data or loss to follow-up. Patients with a history of myocardial infarction within 4 weeks prior to admission were excluded.

### 2.3 Treatment regimens

All patients received conventional comprehensive treatment during hospitalization following the Chinese Guidelines for the Diagnosis and Treatment of Heart Failure 2024 (4), including oxygen therapy, ECG monitoring, diuretics (such as furosemide or torsemide) for volume control, oral or intravenous use of heart failure medications including ACEI/ARB/ARNI, *β*-blockers, and aldosterone receptor antagonists, as well as necessary inotropic agents, glucose control, electrolyte correction, anticoagulation/antiplatelet therapy, and other supportive measures. The specific medications and doses administered in each group were as follows: In the control group, diuretics included furosemide (20–40 mg intravenously or orally, once or twice daily) or torsemide (10–20 mg orally, once daily), adjusted according to fluid status. ACEI/ARB/ARNI agents included enalapril (2.5–10 mg twice daily), valsartan (40–160 mg twice daily), or sacubitril/valsartan (50–200 mg twice daily, initiated at lower doses in elderly patients with systolic blood pressure 100–110 mmHg). *β*-blockers included bisoprolol (1.25–10 mg once daily), carvedilol (3.125–25 mg twice daily), or metoprolol succinate (23.75–190 mg once daily), initiated at low doses and titrated to target or maximum tolerated doses. Aldosterone receptor antagonists included spironolactone (20–40 mg once daily). Inotropic agents (dobutamine or milrinone) were administered when necessary for hemodynamic support. The study group received the same conventional treatment regimen as the control group, with the addition of rhBNP as described below. The study group additionally received rhBNP (Chengdu Nuodikang Bio-Pharmaceutical Co., Ltd., approval number S20050033). The specific administration procedure was as follows: 500 μg rhBNP was dissolved in 50 mL of 0.9% sodium chloride injection. A loading dose of 1.5 μg/kg was administered by intravenous bolus over 3–5 min, followed by continuous intravenous infusion at a maintenance dose of 0.0075–0.015 μg/min for 72 h. During medication administration, patients were continuously monitored for blood pressure, heart rate, oxygen saturation, and urine output; serum electrolytes and renal function were monitored daily. If systolic blood pressure dropped below 90 mmHg or decreased ≥30 mmHg from baseline, the infusion was immediately halved or paused, and fluid resuscitation or medication adjustments were administered.

### 2.4 Observation indicators and detection methods

#### 2.4.1 Clinical therapeutic effects

Markedly effective: clinical symptoms nearly disappeared, and NYHA cardiac function improved by ≥2 classes; Effective: clinical symptoms improved, and NYHA cardiac function improved by 1 class; Ineffective: criteria above not met. Total effective rate = (markedly effective + effective cases)/total cases×100%.

#### 2.4.2 Cardiac function indicators

Pre-treatment, Post-treatment, a color Doppler ultrasound diagnostic system was used to measure left ventricular ejection fraction (LVEF), left ventricular end-diastolic diameter (LVEDD), left ventricular end-systolic diameter (LVESD), and cardiac index (CI).

#### 2.4.3 Blood gas indicators

Pre-treatment blood samples were collected within 24 h of hospital admission before initiation of rhBNP or conventional therapy. Post-treatment blood samples were collected on day 5 after treatment initiation (i.e., 72 h after completion of rhBNP infusion in the study group, or at the corresponding time point in the control group). 5 mL of arterial blood was collected, and arterial oxygen partial pressure (PaO2) and arterial carbon dioxide partial pressure (PaCO2) were measured using a blood gas analyzer.

#### 2.4.4 Myocardial injury markers

Pre-treatment, Post-treatment, 10 mL of fasting venous blood was collected and centrifuged at 3,000 r/min (centrifugal radius 12 cm) for 7 min. The serum was obtained and analyzed using an automated biochemical analyzer to measure N-terminal pro-B-type natriuretic peptide (NT-proBNP), cardiac troponin I (cTnI), lactate dehydrogenase (LDH), and creatine kinase-MB (CK-MB).

#### 2.4.5 Fibrosis and inflammation-related biomarkers

Pre-treatment, Post-treatment, the remaining serum samples were collected. Serum PⅢNP and suPAR concentrations were measured using commercially available enzyme-linked immunosorbent assay (ELISA) kits. PⅢNP was measured using the Human PⅢNP ELISA Kit (Elabscience Biotechnology Co., Ltd., Wuhan, China; catalog number E-OSEL-H0033; sandwich ELISA; detection range: 0.31–20 ng/mL; intra-assay coefficient of variation < 8%, inter-assay CV < 10%). suPAR was measured using the Human suPAR ELISA Kit (Elabscience Biotechnology Co., Ltd., Wuhan, China; catalog number E-EL-H2584; sandwich ELISA; detection range: 0.16–10 ng/mL; intra-assay CV < 8%, inter-assay CV < 10%). A multifunctional microplate reader was used to measure the optical density values of samples and standards at 450 nm, generate standard curves, and calculate serum concentrations.

#### 2.4.6 Adverse reactions and adverse prognosis

All 124 enrolled patients (62 in the control group and 62 in the study group) successfully completed the 3-month follow-up period after discharge, with no losses to follow-up. A 3-month follow-up after discharge was conducted to compare adverse reactions (hypotension, angioedema, hypokalemia, gastrointestinal reactions, etc.) and the occurrence of rehospitalization or all-cause mortality.

### 2.5 Data collection and management

Clinical data were independently extracted from electronic medical records by two researchers and recorded into a standardized data form. Any discrepancies were resolved by a third researcher. Missing data were documented first and then traced by designated personnel; if not retrievable, the cases were excluded.

### 2.6 Statistical analysis

All statistical analyses were performed using SPSS 26.0 (IBM Corp., Armonk, NY, USA), and graphical presentation was generated using GraphPad Prism 8. Normality of continuous variables was evaluated by the Shapiro–Wilk test pre analysis. Normally distributed data were expressed as ($\bar{x}\pm s$), and the independent samples t-test was used for comparisons between groups; paired t-test was used for Pre-treatment and Post-treatment comparisons. For outcome variables with repeated measurements (Pre-treatment and Post-treatment), a two-way repeated-measures analysis of variance (ANOVA) was additionally performed to assess the interaction between time (Pre- vs. Post-treatment) and group (control vs. study), thereby strengthening the validity of the findings. The repeated-measures ANOVA included group as a between-subjects factor and time as a within-subjects factor; the interaction term (group×time) was specifically examined to determine whether changes over time differed between groups. For nonnormally distributed data, the median and interquartile range were used, and the Mann–Whitney U test was performed for between-group comparisons, while the Wilcoxon signed-rank test was used for paired data. Categorical variables were presented as frequencies and percentages; group comparisons were performed using the chi-square test, and Fisher's exact test was used when expected frequencies were insufficient. Given the retrospective nature of the study, baseline characteristics were compared between groups to ensure comparability. Although no significant baseline differences were observed, adjusted analyses were performed for outcome comparisons using analysis of covariance (ANCOVA) with baseline values of the respective outcome variables as covariates, to control for potential residual confounding. Adjusted means and 95% confidence intervals were reported for between-group comparisons. All tests were two-tailed, and statistical significance was set at *P* < 0.05.

## 3 Results

### 3.1 Comparison of baseline characteristics

A total of 162 elderly CHF patients were initially screened between February 2023 and April 2025. After applying the inclusion and exclusion criteria, 38 patients were excluded, and the remaining 124 patients were finally enrolled in this study, with 62 patients in each group. The detailed patient screening process is presented in Figure 1. All 124 patients completed the 3-month follow-up, with no losses to follow-up.

**Figure 1:**
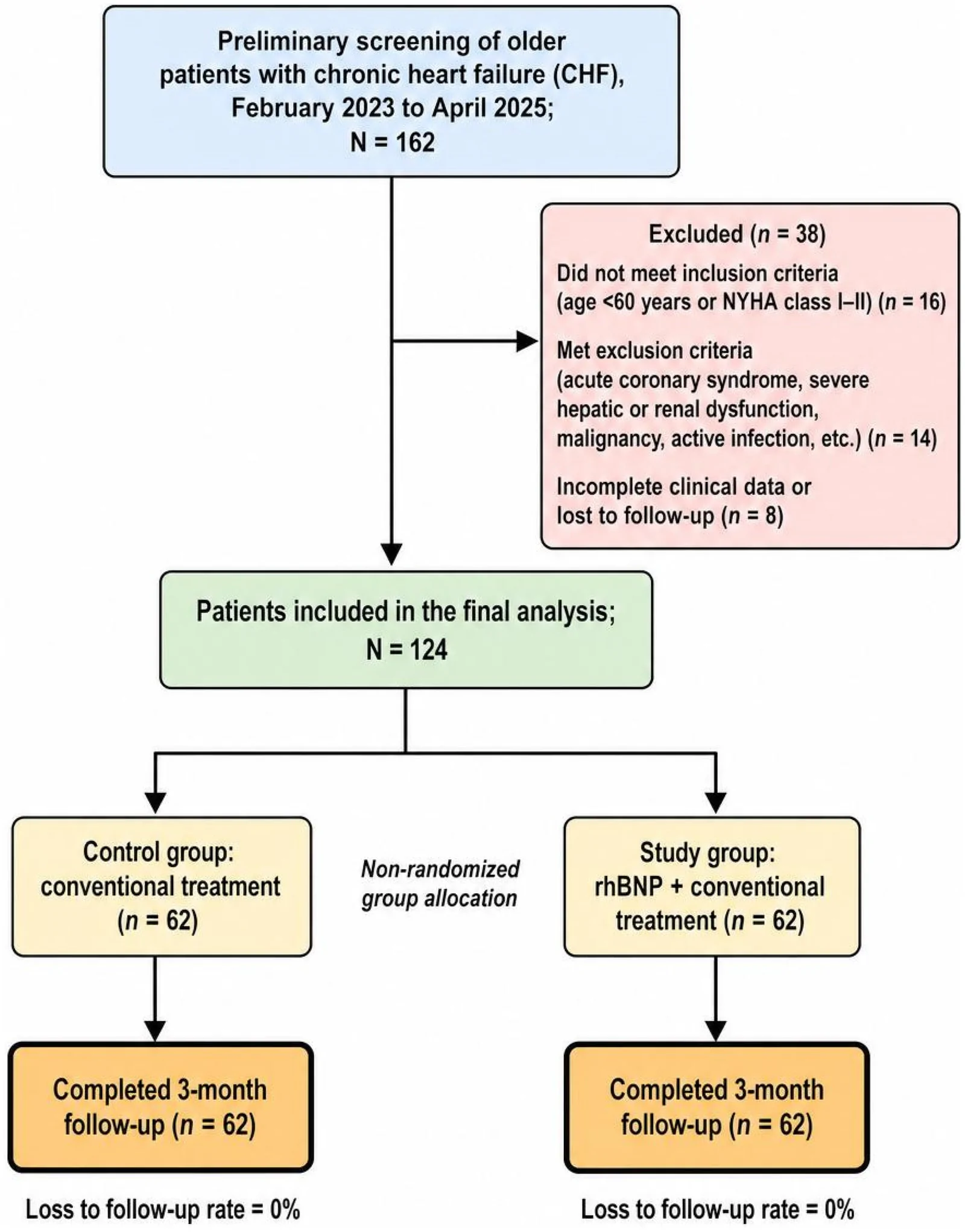
Patient flow diagram.

Of the 124 patients included in the final analysis, there were no significant differences between the two groups in sex, age, NYHA cardiac function classification, primary diseases, and comorbidities (*P* > 0.05), indicating good comparability, as shown in Table 1.

**Table 1:** Comparison of baseline characteristics.

Group (n)	Sex		Age (years)	NYHA cardiac function classification	
	Male	Female		III	IV
Control (*n* = 62)	40	22	64.82 ± 9.74	42	20
Study (*n* = 62)	36	26	66.13 ± 10.21	45	17
* t/x^2^*	0.543		0.731	0.346	
* P*	0.460		0.466	0.556	

Group (n)	Primary diseases				Comorbidities		
	Coronary heart disease	Hypertensive heart disease	Dilated cardiomyopathy	Other	Diabetes	Hypertension	Hyperlipidemia
Control (*n* = 62)	28	14	10	10	22	35	18
Study (*n* = 62)	31	12	7	12	25	38	15
* x^2^*	0.291				0.308	0.299	0.371
* P*	0.589				0.578	0.584	0.542

### 3.2 Comparison of clinical therapeutic effects

The overall therapeutic efficacy of the study group Post-treatment was significantly better than that of the control group (total effective rate: 93.55% vs. 82.26%, P＜0.05), as shown in Figure 2.

**Figure 2:**
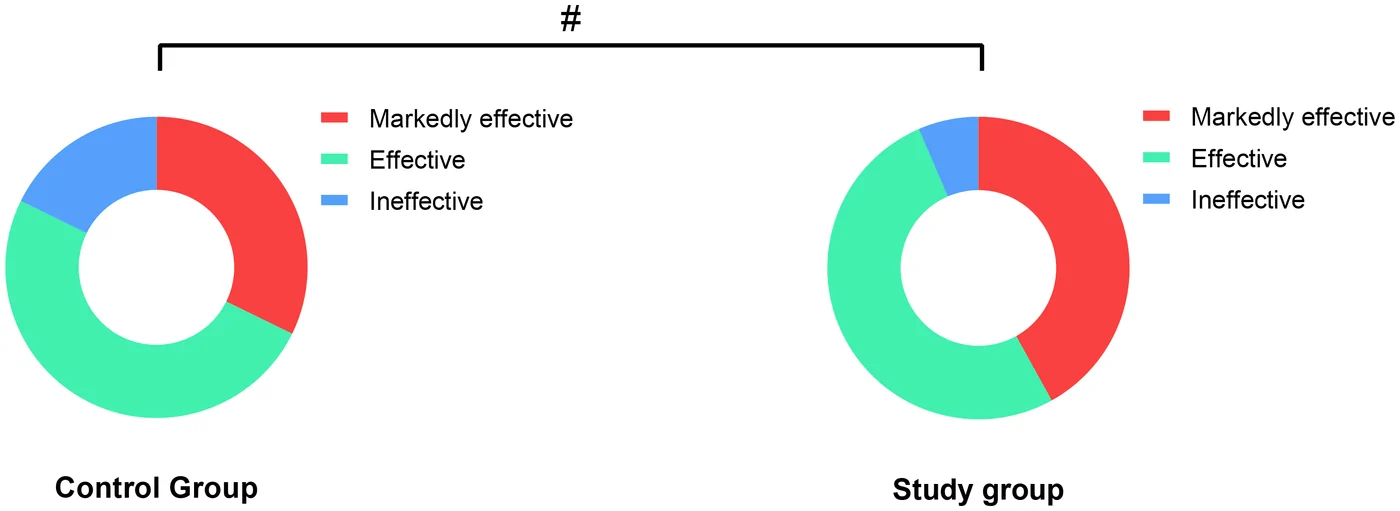
Comparison of clinical therapeutic effects. Compared between groups, ^#^P<0.05.

### 3.3 Comparison of cardiac function indicators

Post-treatment, LVEF and CI increased in both groups compared with Pre-treatment, whereas LVEDD and LVESD decreased. The degree of improvement was more significant in the study group (P＜0.05), as shown in Figure 3. Repeated-measures ANOVA revealed a significant group×time interaction for LVEF (F = 8.42, *P* < 0.001), LVEDD (F = 7.96, *P* < 0.001), LVESD (F = 7.53, *P* < 0.001), and CI (F = 6.87, *P* < 0.001), indicating that the improvements over time were significantly greater in the study group than in the control group. ANCOVA adjusted for baseline values confirmed these between-group differences (all *P* < 0.05).

**Figure 3:**
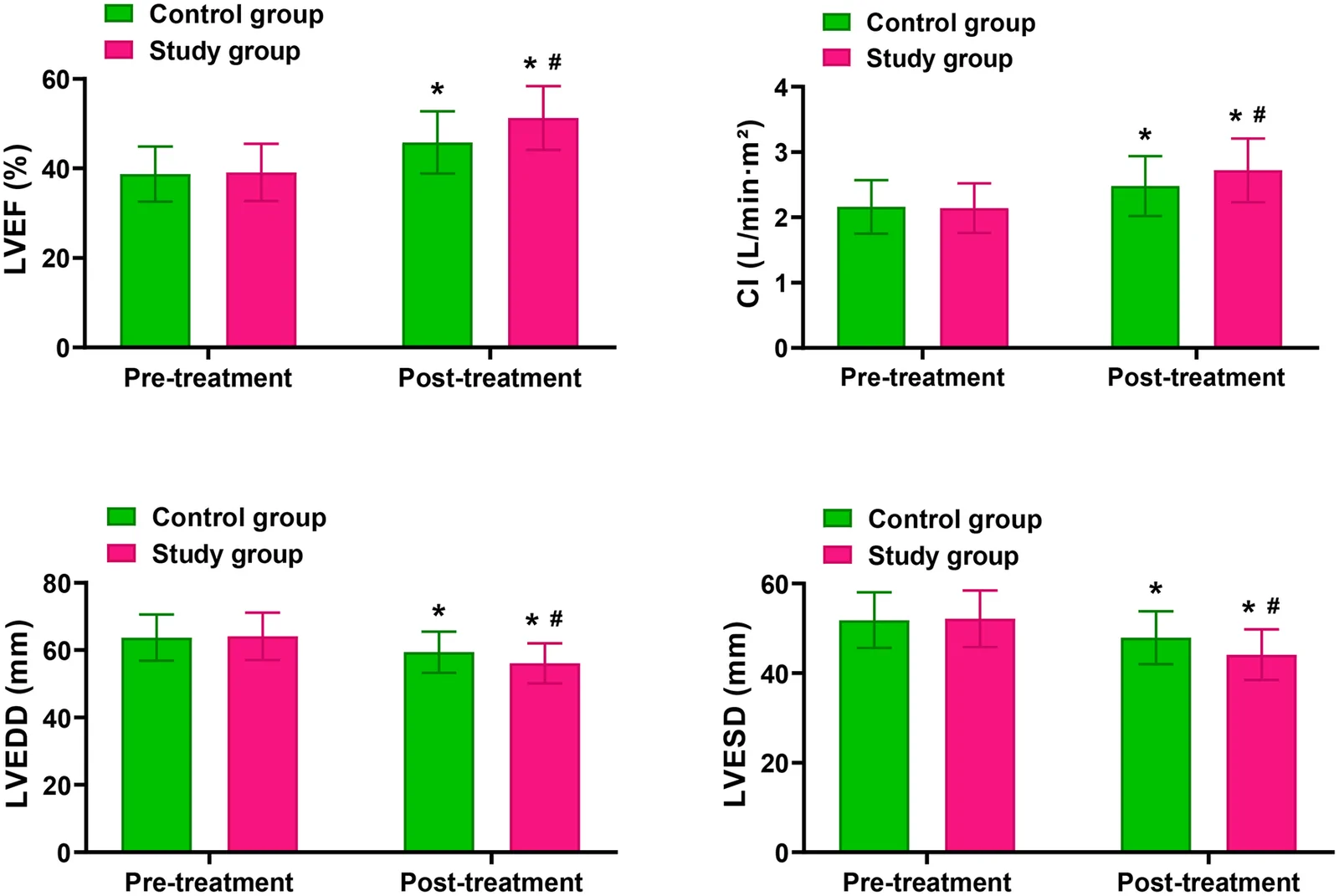
Comparison of cardiac function indicators. Compared with Pre-treatment in the same group, *P<0.05; compared between groups, ^#^P<0.05.

### 3.4 Comparison of blood gas indicators

Post-treatment, PaO2 increased and PaCO2 decreased in both groups compared with Pre-treatment, with greater improvements in the study group (P＜0.05), as shown in Table 2. Repeated-measures ANOVA confirmed significant group×time interactions for PaO2 (F = 6.53, *P* < 0.001) and PaCO2 (F = 5.89, *P* < 0.001).

**Table 2:** Comparison of blood Gas indicators (mmHg).

Group (n)	PaO2		PaCO2	
	Pre-treatment	Post-treatment	Pre-treatment	Post-treatment
Control (*n* = 62)	69.41 ± 7.85	81.26 ± 8.14*	46.32 ± 5.78	41.28 ± 5.31*
Study (*n* = 62)	69.92 ± 8.02	88.37 ± 8.53*	46.51 ± 6.03	38.12 ± 4.88*
* t*	0.357	4.748	0.179	3.450
* P*	0.721	＜0.001	0.858	＜0.001

Compared with Pre-treatment in the same group, *P＜0.05.

### 3.5 Comparison of myocardial injury indicators

Post-treatment, NT-proBNP, cTnI, LDH, and CK-MB levels in both groups decreased compared with Pre-treatment, with greater reductions in the study group (P＜0.05), as shown in Figure 4. Repeated-measures ANOVA demonstrated significant group×time interactions for all four markers (NT-proBNP: F = 9.14, *P* < 0.001; cTnI: F = 7.22, *P* < 0.001; LDH: F = 6.45, *P* < 0.001; CK-MB: F = 6.08, *P* < 0.001).

**Figure 4:**
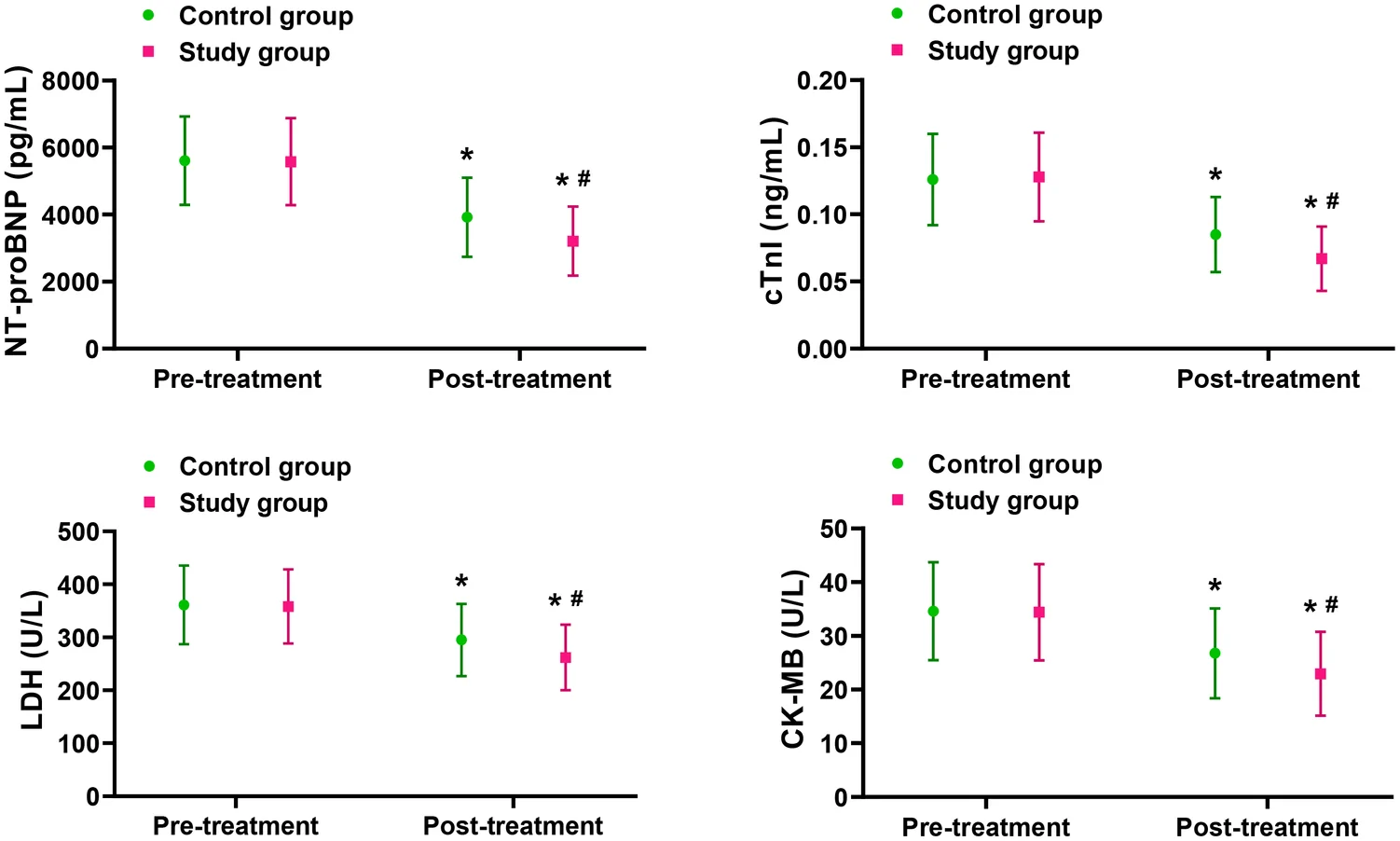
Comparison of myocardial injury indicators. Compared with Pre-treatment in the same group, *P<0.05; compared between groups, ^#^P<0.05.

### 3.6 Comparison of fibrosis and inflammation-related biomarkers

Post-treatment, PⅢNP and suPAR levels decreased in both groups compared with Pre-treatment, with greater reductions in the study group (P＜0.05), as shown in Table 3. Repeated-measures ANOVA revealed significant group×time interactions for PⅢNP (F = 8.31, *P* < 0.001) and suPAR (F = 7.95, *P* < 0.001).

**Table 3:** Comparison of fibrosis and inflammation-related biomarkers (ng/mL).

Group (n)	PⅢNP		suPAR	
	Pre-treatment	Post-treatment	Pre-treatment	Post-treatment
Control (*n* = 62)	10.84 ± 2.21	8.42 ± 2.05*	4.62 ± 1.03	3.78 ± 0.96*
Study (*n* = 62)	10.76 ± 2.18	7.01 ± 1.76*	4.58 ± 1.07	3.12 ± 0.84*
* t*	0.202	4.109	0.212	4.074
* P*	0.839	＜0.001	0.832	＜0.001

Compared with Pre-treatment in the same group, *P＜0.05.

### 3.7 Comparison of adverse reactions and adverse prognosis

During the 3-month follow-up period, all patients successfully completed the follow-up with no losses. There were no significant differences between the two groups in the incidence of adverse reactions or adverse prognostic events (P＞0.05), as shown in Table 4.

**Table 4:** Comparison of adverse reactions and adverse prognosis.

Group (n)	Adverse reactions				Adverse prognosis	
	Hypotension	Angioedema	Hypokalemia	Gastrointestinal reactions	All-cause mortality	Readmission
Control (*n* = 62)	6	2	6	11	5	13
Study (*n* = 62)	9	1	4	8	3	9
* x^2^*	0.682	0.000	0.435	0.559	0.133	0.884
* P*	0.408	1.000	0.509	0.454	0.714	0.347

## 4 Discussion

This study systematically evaluated the efficacy and changes in biological markers in elderly CHF patients treated with rhBNP on the basis of conventional therapy. The results showed that the study group exhibited more significant improvements than the control group in overall clinical efficacy, cardiac function, blood gas parameters, myocardial injury markers, and fibrosis/inflammation-related biomarkers (PⅢNP, suPAR), while no significant differences were observed in adverse reactions and short-term adverse outcomes between the two groups. Regarding clinical efficacy and improvement in cardiac function, the present study showed that the addition of rhBNP to standard therapy significantly increased the total effective rate and led to more pronounced improvements in LVEF and CI, as well as greater reductions in LVEDD and LVES. These findings can be reasonably explained by the pharmacological actions of rhBNP: rhBNP mimics endogenous BNP, reducing cardiac preload and afterload through venous and arterial dilation, promoting diuresis and natriuresis to lower cardiac burden, and inhibiting excessive activation of the RAAS and sympathetic nervous system to alleviate myocardial stress, thereby improving hemodynamic status in the short term and contributing to the reversal of ventricular remodeling (16–18). The greater improvements in LVEF and CI observed in the study group support the notion that rhBNP not only alleviates symptoms and hemodynamic abnormalities but may also help restore myocardial function by reducing myocardial wall stress and improving myocardial perfusion. This is consistent with previous studies reporting rapid hemodynamic improvements after BNP administration in acute decompensated heart failure (19, 20). The present study extends these benefits to elderly CHF patients, suggesting that rhBNP may enhance existing therapies particularly in elderly patients with unstable conditions or recent exacerbations.

The significant improvement in blood gas parameters (PaO2, PaCO2) observed in the study group indicates that rhBNP combined therapy also exerts beneficial effects on systemic oxygenation and respiratory metabolic status. HF patients often develop hypoxemia or CO₂ retention due to pulmonary congestion, ventilation/perfusion imbalance, or coexisting COPD (21, 22). rhBNP reduces pulmonary circulatory pressure, alleviates pulmonary congestion, and improves left ventricular diastolic and systolic function (23), thereby enhancing alveolar-capillary gas exchange, leading to elevated PaO2 and reduced PaCO2. The findings of this study align with previous clinical observations (24, 25), indicating that improvements in cardiovascular loading conditions can concurrently enhance oxygenation—a particularly crucial benefit for elderly patients who have reduced tolerance to hypoxia and higher complication risks.

The significant reductions in myocardial injury markers (NT-proBNP, cTnI, LDH, CK-MB), with more pronounced changes in the study group, reflect the potential of rhBNP to reduce myocardial stress and cellular injury. NT-proBNP, as a sensitive indicator of ventricular wall stress and HF severity, decreases in association with reduced cardiac burden and improved prognosis (26). Decreases in cTnI, LDH, and CK-MB may reflect a reduction in ischemic or stress-related myocardial injury (27). Mechanistically, by reducing cardiac preload and afterload, improving coronary perfusion pressure gradients, and inhibiting damaging neurohormonal pathways, rhBNP may mitigate ischemia-reperfusion injury and inflammation-mediated myocardial cell damage. These findings correspond with previous research (28, 29) showing short-term benefits of BNP therapy on myocardial biomarkers. The present study demonstrates similar biological effects in elderly CHF patients, suggesting potential benefit for this specific population.

Another key finding of this study is the more pronounced reduction in PⅢNP and suPAR levels in the study group. PⅢNP, an indicator of type III procollagen metabolism, reflects myocardial fibrosis activity (30); suPAR is considered a stable marker of chronic inflammation and immune activation (31), both associated with ventricular remodeling, functional decline, and poor prognosis. The reduction in PⅢNP suggests that rhBNP may inhibit or slow myocardial fibrosis progression in the short term. Possible mechanisms include suppression of RAAS activity, reduced expression of profibrotic factors (e.g., indirect influence on the TGF-*β* pathway), and decreased activation of cardiac interstitial fibroblasts (32, 33). The reduction in suPAR suggests an alleviation of systemic inflammation and immune activation, potentially due to rhBNP-mediated inhibition of inflammatory mediator release, improved tissue perfusion, and reduced chronic stress (34). Although the present study is limited to short-term observation, the concurrent reductions in PⅢNP and suPAR provide preliminary clinical evidence to support further exploration of the effects of rhBNP on myocardial remodeling and inflammatory pathways. Compared with other studies observing changes in fibrosis or inflammatory markers following HF treatment, this study concurrently noted reductions in both categories within the same population, enhancing the internal consistency of the findings. Lastly, regarding safety, no significant increases in overall adverse reactions or short-term adverse outcomes were observed in the combined treatment group. This is clinically meaningful in the elderly population, who often have multiple comorbidities and reduced medication tolerance; any additional therapy must balance efficacy with safety. Although rhBNP may cause hypotension or other hemodynamic side effects, strict monitoring and timely dose adjustments in this study helped prevent severe adverse events, suggesting that adherence to standardized protocols allows rhBNP to offer additional benefits with manageable risks.

Despite these encouraging findings, the study must be interpreted cautiously in light of its limitations. First, as a retrospective comparative cohort study, it is subject to potential selection and information biases. Although baseline characteristics were comparable and adjusted analyses were performed to control for measured confounders, unmeasured confounders—such as subtle differences in concomitant medications, clinical status fluctuations at admission, physician treatment preferences, or socioeconomic factors—may still influence outcomes. Second, the lack of randomization means that treatment assignment was determined by clinical judgment rather than by a randomized allocation mechanism, which may introduce treatment-selection bias. Although the two groups were comparable in baseline characteristics, the possibility of confounding by indication cannot be entirely excluded. Third, the sample size of 62 patients per group, while determined by power calculation, remains relatively modest; larger studies would be needed to detect smaller effect sizes or subgroup differences. Fourth, the follow-up duration of 3 months is relatively short, limiting the ability to assess long-term outcomes such as 1-year rehospitalization rates, cardiovascular mortality, or sustained improvements in quality of life. Longer follow-up is needed to determine whether the observed short-term benefits translate into durable clinical advantages. Fifth, variability in rhBNP dosage, timing, and duration inherently exists in retrospective data. Although patients were included within a standard dose range, individualized adjustments were unavoidable and may affect effect estimates. Sixth, biomarkers such as PⅢNP and suPAR are influenced by multiple factors (renal function, chronic inflammation, metabolic diseases, etc.). Despite rigorous inclusion and exclusion criteria and adjusted analyses, residual confounding cannot be fully eliminated. Therefore, the study's conclusions should be interpreted as associations rather than causal evidence. Future research should proceed along two directions: prospective, multicenter, randomized controlled trials to validate the efficacy and safety of rhBNP in elderly CHF patients, with standardized dosing protocols, strict follow-up, and long-term outcome evaluation (e.g., 1-year rehospitalization rate, cardiovascular mortality, quality-of-life scores); and mechanistic studies incorporating cardiac imaging (such as cardiac MRI for fibrosis assessment), histology, and molecular analyses to clarify rhBNP's effects on myocardial fibrosis pathways, inflammatory signaling, and metabolic profiles. Additionally, investigating whether changes in PⅢNP and suPAR mediate the functional improvements associated with rhBNP treatment may provide a biomarker-based foundation for individualized therapeutic strategies.

## 5 Conclusion

This retrospective single-center study provides preliminary evidence that rhBNP combined with conventional therapy may offer potential benefits for elderly CHF patients, particularly in improving cardiac function, blood gas status, myocardial injury markers, and fibrosis/inflammation biomarkers. However, given the limitations of the study design, these findings warrant further verification through higher-level evidence. Future randomized controlled trials and mechanistic studies will help clarify the role of rhBNP in CHF management and guide its precise and safe clinical application, ultimately contributing to improved prognosis and quality of life for elderly CHF patients.

## Data Availability

The original contributions presented in the study are included in the article/Supplementary Material, further inquiries can be directed to the corresponding author.
